# Signal Amplification in Electrochemical DNA Biosensors Using Target-Capturing DNA Origami Tiles

**DOI:** 10.1021/acssensors.2c02469

**Published:** 2023-03-13

**Authors:** Paul Williamson, Petteri Piskunen, Heini Ijäs, Adrian Butterworth, Veikko Linko, Damion K. Corrigan

**Affiliations:** † Department of Biomedical Engineering, University of Strathclyde, Glasgow G1 1QE, United Kingdom; ‡ Biohybrid Materials, Department of Bioproducts and Biosystems, Aalto University, 00076 Aalto, Finland; § Ludwig-Maximilians-University, Geschwister-Scholl-Platz 1, 80539 Munich, Germany; ∥ LIBER Center of Excellence, Aalto University, 00076 Aalto, Finland; ⊥ Institute of Technology, University of Tartu, Nooruse 1, 50411 Tartu, Estonia; # Department of Pure & Applied Chemistry, Thomas Graham Building, University of Strathclyde, 295 Cathedral Street, Glasgow G1 1XL, United Kingdom

**Keywords:** DNA nanotechnology, DNA hybridization, electrochemical impedance spectroscopy, antimicrobial resistance gene, target selectivity, sensitivity enhancement, point-of-care devices

## Abstract

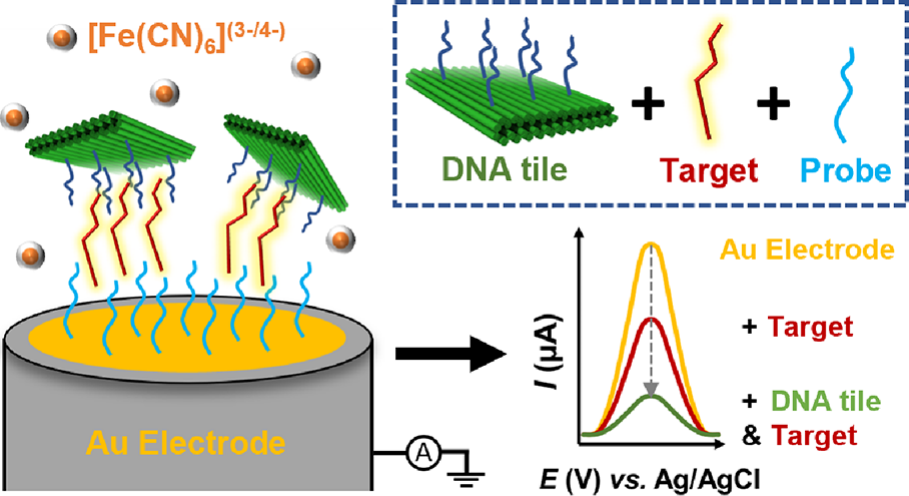

Electrochemical DNA (e-DNA) biosensors are feasible tools for disease monitoring, with their ability to translate hybridization events between a desired nucleic acid target and a functionalized transducer, into recordable electrical signals. Such an approach provides a powerful method of sample analysis, with a strong potential to generate a rapid time to result in response to low analyte concentrations. Here, we report a strategy for the amplification of electrochemical signals associated with DNA hybridization, by harnessing the programmability of the DNA origami method to construct a sandwich assay to boost charge transfer resistance (*R*_CT_) associated with target detection. This allowed for an improvement in the sensor limit of detection by two orders of magnitude compared to a conventional label-free e-DNA biosensor design and linearity for target concentrations between 10 pM and 1 nM without the requirement for probe labeling or enzymatic support. Additionally, this sensor design proved capable of achieving a high degree of strand selectivity in a challenging DNA-rich environment. This approach serves as a practical method for addressing strict sensitivity requirements necessary for a low-cost point-of-care device.

Electrochemical DNA biosensors hold significant promise for the monitoring of various diseases. Central to their efficacy is the inherent strict base pair binding of DNA, allowing for highly efficient hybridization between complementary sequences. With the immobilization of single-stranded probe oligonucleotides into a self-assembled monolayer, a transducer surface (planar gold or carbon electrodes) can be functionalized to capture targets with high selectivity. Employing a supportive background electrolyte and redox species in solution, it is possible to measure variations in electrochemical processes, associated with DNA binding events across an electrode surface.

The potential applications are vast, with target analytes ranging from bacterial nucleic acids associated with antimicrobial resistance (AMR),^1−3^ circulating tumor DNA sequences (ctDNA),^4,5^ single nucleotide polymorphisms,^6,7^ and recent detection of clinically relevant concentrations of biomarkers for SARS CoV-2 with aptasensors.^8^ This method of target analyte detection has proven successful in various laboratory-based setups. Despite these advances, translation of such systems from the laboratory to a clinical environment is yet to occur and yield the diagnostic revolution often promised. There are numerous issues yet to be resolved, associated with DNA biosensing at the fundamental level. Much of this stems from the inherent variability in the manufacture of uniform DNA layers, self-assembled monolayer (SAM) instability, required optimization of receptor molecule packing densities, availability of binding sites for target hybridization, and the determination of appropriate electrochemical parameters for maximum signal gain.^8−15^ This has contributed to multiple novel solutions for controlling packing densities of immobilized probes,^16,17^ often incorporating the use of amplification strategies for detection of low concentration analytes,^18,19^ or the improving target hybridization efficiency with peptide nucleic acid (PNA) analogues.^20^ However, these approaches often involve complex chemistries, electrochemical labeling, technically challenging materials, or multistep processing. Ultimately, this generates impressive sensitivity limits, though pushes potential applications beyond the scope of a low-cost point-of-care (PoC) device.

Meanwhile, structural DNA-based nanotechnology,^21,22^ especially the DNA origami technique,^23−25^ offers almost unrivaled spatial control over target molecules of interest. In DNA origami, a long single-stranded DNA scaffold is self-assembled into a user-defined shape upon mixing and annealing with short synthetic staple strands. The structures can then be further modified with customizable binding sites, coatings, or other components as desired.^25^ This facilitates easy assembly of even complex nanoscale shapes for all manners of purposes, such as the templating of other materials for, e.g., materials science^26^ and nanoelectronics,^27^ controlled and targeted drug delivery,^28,29^ nanorobotics,^30,31^ gene editing,^32^ and sensing^33^ to name a few. The key capabilities of DNA origami lie in their modular nature and the addressability of each individual nucleobase in their structures, which enable accurate and reliable subnanometer positioning of functional elements like target molecules,^34^ proteins,^35^ or optically active particles.^36,37^ These qualities make DNA origami a versatile and promising pathway also for enhancing various measurement^38−40^ and biosensing tools.^33,41−43^

The current applications of DNA origami in biosensing primarily focus on the optimization of capture element positioning for the electrochemical detection of simple nucleic acids,^44^ large synthetic mesoscale targets,^45^ or the voltage-driven, single-molecule capture of proteins in a nanopore.^46^ Recently, also, electrically actuated DNA origami nanolevers^47,48^ and zippers^40^ have been coming increasingly into view. Such structures have lately also been investigated in terms of their environment- and structure-dependent behavior.^49,50^ In addition to being able to actuate the DNA origami levers with electrical inputs for various uses, the levers themselves can also conversely modulate the electrical properties of the surfaces that they are bound to, enabling their use as electrochemical sensing elements.

Following this line of thought, here, we report on an approach for DNA detection limit amplification using programmable DNA origami tiles. The pegboard-like DNA origami serves as a simple and modular platform for the target-dependent tethering of the tile to a functionalized electrode (FE) (probes + polycrystalline gold electrode (PGE)) (Figure 1). Incorporating these nanostructure assemblies to a conventional Faradaic, label-free electrochemical biosensor methodology, it is possible to achieve significant improvements in detection capabilities and shift the linear working range of a sensor to the low pM range, with a high strand degree of selectivity. Importantly, the translation of such a sensing design to low-cost, disposable thin-film gold electrodes positions this new technology with a route to mass manufacturability and broad applicability.

**Figure 1:**
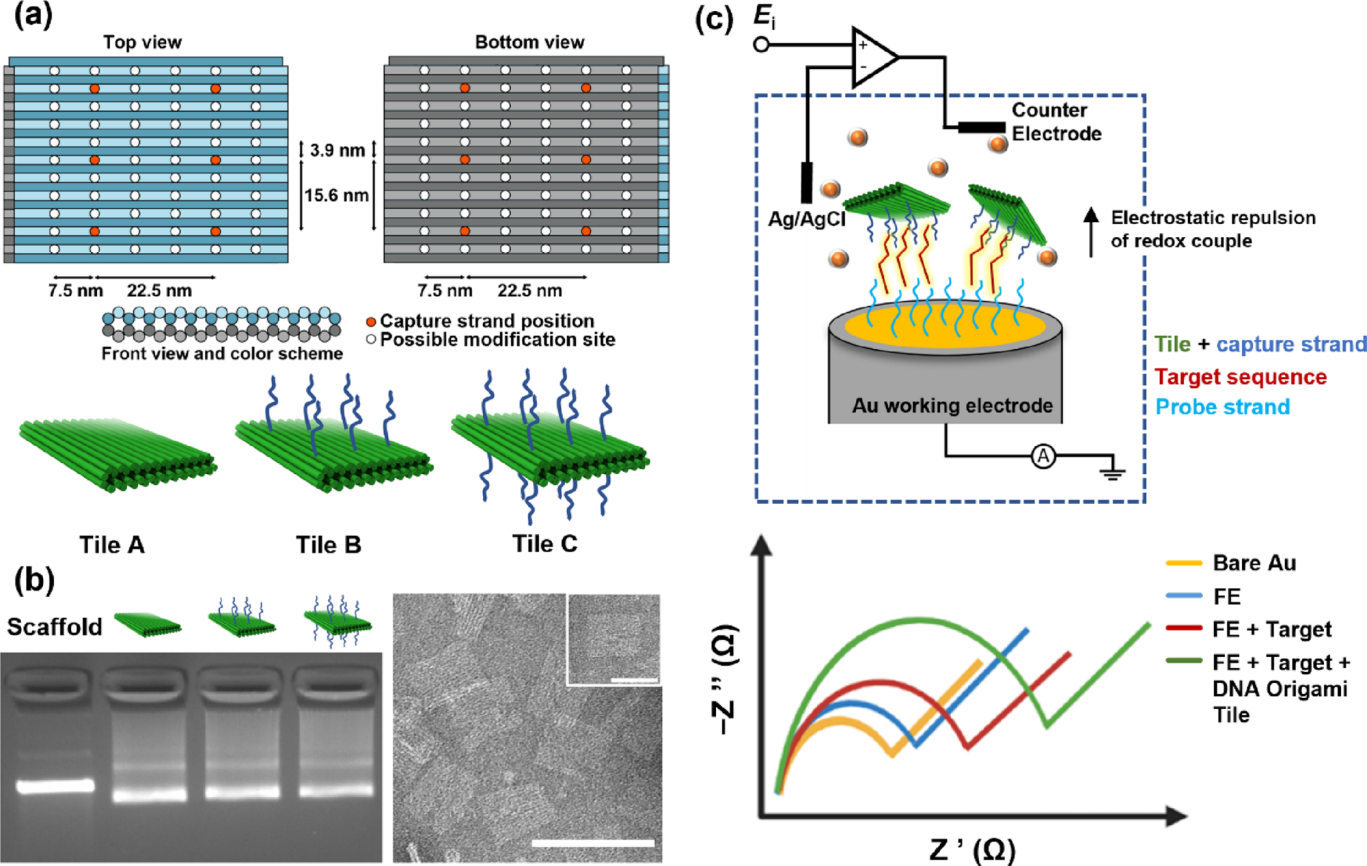
Design and characterization of the DNA origami tiles and their use in signal amplification in biosensors. (a) Top, bottom, and front views of the pegboard-like DNA origami tile design with possible modification sites (white circles) and the selected locations for capture strand modifications (red circles). Schematic view of the three different tiles: tile A, B, and C with 0, 6, and 12 capture strands (deep blue), respectively. (b) Left: agarose gel electrophoresis analysis for the folded DNA origami tiles shown in (a). The bands shift slightly corresponding to added capture strands. Right: transmission electron microscopy (TEM) images of the DNA origami design. The scale bars are 50 and 100 nm for the inset and large-scale image, respectively. (c) Hypothesis of the signal amplification in the biosensor through the implementation of DNA origami tiles. Top panel: DNA origami (green) with capture strands (deep blue) bind to the target strands (red), and the formed complex further attaches to the ssDNA-probe (light blue)-functionalized electrode (FE) (probes + polycrystalline gold electrode (PGE)), thus modulating the distribution of the redox species. Bottom panel: schematic electrochemical impedance spectroscopy (EIS) responses. EIS can be used to monitor the drastic increase in the charge transfer resistance (*R*_CT_) as the target-capturing DNA origami tile is present.

## Materials and Methods

### DNA Origami Design and Characterization

The basic DNA origami tile was designed using caDNAno,^51^ and it is based on a previously published two-layered honeycomb-lattice DNA origami pegboard.^52^ The plate-like design features 66 evenly spaced modification sites with 3.9 nm × 7.5 nm separations on both sides in identical positions (in total 132 binding sites). For this study, 0, 6, or 12 sites were used for creating extended capture strands. In other words, we used three versions of the tile design with either 0 (tile A), 6 (tile B, strands on one side), or 12 capture strands (tile C, 6 strands per each side) (see Figure 1a). To create the different tile versions, individual core staples at the modification sites were replaced by staples with capture strand extensions added to their 3′ ends (5′-ttttttTTGTCTTCGTACCGAGCTTTCATCGAATTTTTA-3′, where “tttttt” denotes a poly-T_6_ spacer sequence). Details of DNA origami-related materials, tile designs (Supporting Information Figure S1), replaced strand sequences, the assembly and purification protocols, as well as the characterization of the ready structures (agarose gel electrophoresis (AGE)) (Figure 1b), and transmission electron microscopy (TEM) (Figure 1b and Figures S2–S4) are described in the Supporting Information.

### Sensor Construction, Probes, Targets and Other Materials

All electrochemical measurements were undertaken using an Autolab PGSTAT128N potentiostat with the additional FRA32M electrochemical impedance spectroscopy module, by scripts written in the Nova 2.1 software package (Metrohm Autolab). PGEs of a 2 mm diameter were purchased from IJ Cambria Scientific Ltd. (Llanelli, UK). An external platinum counter electrode (Metrohm, Runcorn, UK) and a Ag/AgCl 3 M KCl reference electrode (Cole-Parmer, UK) completed the electrochemical cell. Here, immobilized probes, and capture strands are primer sequences for the amplification of a region of an artificial plasmid attributing to the *bla*OXA-1 β-lactamase gene; encoding extended-spectrum β-lactamases (ESBLs) and resistance to Oxacillin, across a host of gram-negative species. This *bla*OXA-1 β-lactamase gene sequence (115 nt OXA fragment) serves as the complementary target sequence in this study. All oligonucleotides for sensor construction were sourced from Sigma Aldrich (Dorset, UK) (given in Supporting Information Table S1). 3-Mercapto-1-propanol (MCP) and all other chemicals required in this study were obtained from Sigma Aldrich (Dorset, UK). Buffers required in this work are detailed in Supporting Information Table S2. Details of electrode preparation, functionalization, electrochemical measurement, and target incubation are available in the Supporting Information.

## Results and Discussion

### Electrode Functionalization and Detection of the Free Target

**Figure 2:**
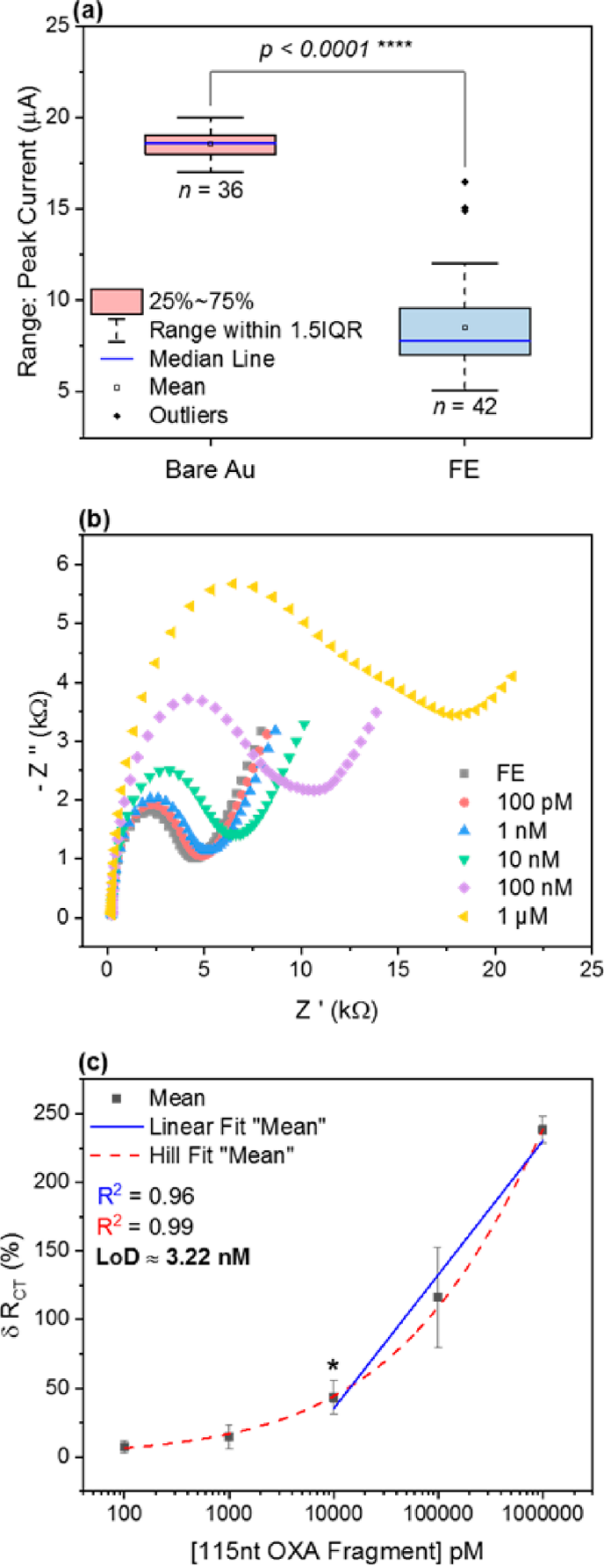
Electrochemical characterization of SAM assembly and sensing performance without DNA origami tile amplification. (a) Comparison of mean peak current (μA) for cleaned PGE (bare Au) and functionalized electrodes (FE). (b) Mean EIS signal response to varying concentrations of the complementary target (115 nt OXA fragment). (c) Mean percentage change in *R*_CT_ plotted against a varying concentration of the complementary target. *n* = 4 PGE for both (b) and (c). Error bars = SD.

Prior to assessing the enhancement of sensing performance by DNA origami tiles, it was then necessary to characterize the functionalization of gold electrode surfaces. In this study, polycrystalline gold electrodes were selected because of the ability to clean in piranha solution (to remove organic contaminants) and to regenerate these surfaces with high repeatability using standard electrode polishing techniques. In order to assess the immobilization behavior of the DNA probe as part of a mixed SAM, an experiment was carried out where both differential pulse voltammetry (DPV) and electrochemical impedance spectroscopy (EIS) at an open-circuit potential were performed in potassium ferri/ferrocyanide solutions. Potassium ferri/ferrocyanide ([Fe(CN)_6_]^(3–/4−)^) is a commonly employed redox couple for the measurement of DNA immobilization on electrode surfaces. The ferri- and ferrocyanide species possess trivalent and quadrivalent anions, meaning that the interaction with immobilized DNA (a polyanion) is governed by electrostatic repulsion at an electrode surface. Figure 2a details the functionalization process with comparisons drawn between the immobilized ssDNA probe as part of the mixed pDNA/MCP SAM and a pristine electrode surface. Thereafter, Figure 2b,c reports on the capability of functionalized electrodes to monitor the hybridization of free targets without amplification by an origami tile complex.

Figure 2a highlights the reproducibility of both the cleaning and functionalization methodologies for PGE. Mean peak currents from a high sample size of PGE exist with a high degree of significance between them, representative of the SAM forming process. Probe surface densities have been estimated for the functionalization protocol by chronocoulometric methods (Supporting Information Table S3).^13,53^ Adoption of these methods produces a surface coverage of 4.62 ± 2.28 × 10^12^ molecules/cm^2^. This is in good accordance with the literature where a strong hybridization activity is measured electrochemically^13^ and by surface plasmon resonance (SPR) for probe coverages in this range.^54^

In Figure 2b, averaged EIS signal traces in response to varying target (115 nt OXA fragment) concentrations are displayed. Incubation with an increasing concentration contributes to a growth in the semicircle region dominating the medium to a high-frequency range. Figure 2c provides a mean percentage change of *R*_CT_ derived from the EIS traces, plotted against the increasing target concentration. Experimental data are well-fitted by a standard Hill equation for specific binding, with a strong coefficient of correlation at 0.99. The Hill equation employed is as follows:
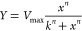
1where *V*_max_ is the maximum binding obtained, *x* is the concentration of the target, *k* is the dissociation coefficient, and *n* is the Hill slope describing cooperativity. The first significant mean percentage change in *R*_CT_ is reported following incubation with 10 nM of a target (*p* = 0.012), which serves as the lower limit of the linear working range of the sensor. Note that the upper limit of the working range for this sensor design is not yet clear, as no saturation point has been achieved. This linear range is shown here by the blue trace, with a correlation coefficient of 0.96. Other approaches to linear fitting could have been employed such as two linear ranges, but the fit was included here for illustrative purposes only.

The limit of detection (LoD) can then be estimated by the following equation:
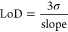
2where σ is the standard deviation of the blank (FE condition) and slope is the Hill slope from the fitting function.^55^ This generates an indicative LoD for this conventional pDNA biosensor design at 3.22 nM.

### Determining an Optimal Origami Tile Design for Signal Amplification

Three origami tile structures were assembled to test the hypothesis of DNA nanostructures inducing the amplification of electrochemical signal change associated with target detection. Direct comparisons were drawn of δ peak current (μA) and *R*_CT_ (Ω) of functionalized electrodes following incubation with DNA origami tiles (tile A, B, or C) at a fixed concentration of 1 nM and the complementary target (115 nt OXA fragment) at 1 nM. EIS is a sensitive and label-free method for probing interfacial parameters, obtaining kinetic information, and monitoring mass transport-limited processes at modified electrode surfaces. In this technique, a small AC potential signal is applied at the working electrode, and the resulting current response is measured. This is performed over a range of frequencies and allows parameters such as the solution resistance (*R*_S_), the double-layer capacitance (*C*_dl_), and the charge transfer resistance (*R*_CT_) to be extracted. It therefore serves as an effective tool for the assessment of this sensing approach.

**Figure 3:**
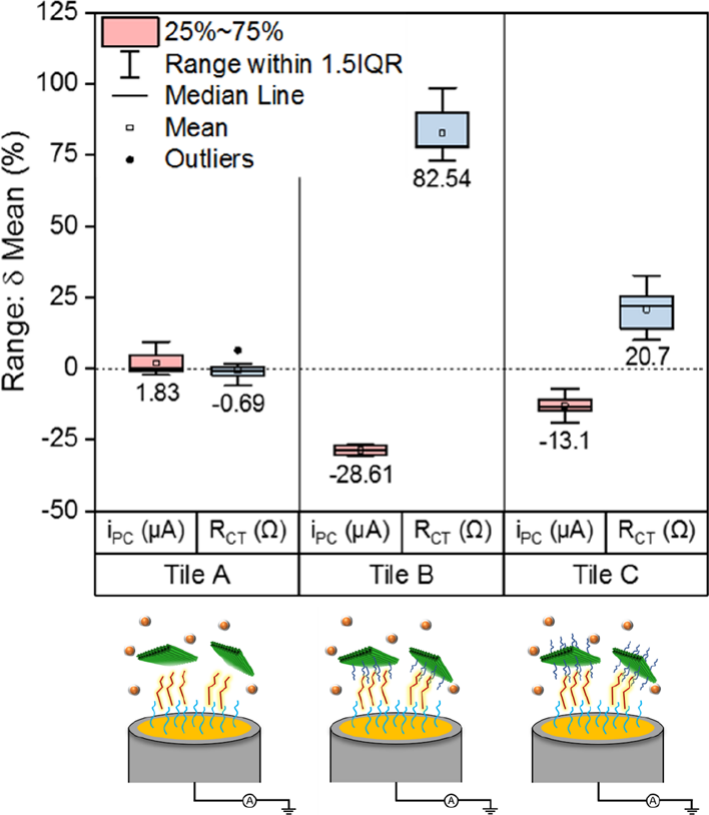
Selection of an appropriate tile design. Mean percentage change of peak current (*i*_PC_) and *R*_CT_ is provided following incubation of tile A, B, or C at 1 nM with the complementary target (115 nt OXA fragment) at 1 nM. *n* = 4 PGE with duplicate measurement per condition. Error bars = SD.

In Figure 3, the ability of various tile designs to hybridize with the complementary target at a matched concentration is reported. The subsequent capture of the resultant complexes by the immobilized probe sequences on the electrode allows for direct comparison of a pretarget/post-target condition. The sensor design incorporating tile A, without capture strands, elicits no significant change in either *i*_PC_ or *R*_CT_. At a target concentration of 1 nM, the underlying probe is not at a sufficient concentration for its hybridization to the immobilized probe to be detected by current electrochemical methods without subsequent amplification. It cannot be confirmed at this stage if the tile A and the target have a method of interaction that is not yet understood. It may be possible to confirm if this is the case with a repeat of this experiment at a significantly higher concentration at 1 μM. Should there be no nonspecific interactions of target and tile A, the expected electrochemical data would be in accordance with that gathered for 1 μM of Figure 2b.

Tile B reports highly significant signal changes for both *i*_PC_ and *R*_CT_. This supports the theory of large origami structures contributing to dramatic manipulation of the interfacial properties for functionalized electrodes via direct tethering through its complementary target present in solution. The impact of amplification by an origami tile is clear when contrasting the mean signal change of a conventional DNA biosensor design to a complementary target against that of our sensor design.

Tile C matches the level of significance in the signal change for that of tile B; however, the magnitude of change is lesser. This suggests that the larger number of target capture strands may boost the number of target sequences occupying both planes of the tile. However, this does not directly aid in the subsequent arrest of this complex by the immobilized probe. As such, tile B was chosen for further interrogation. Signal changes, and the level of significance, for each tile design are reported in Table 1.

**Table 1:** Tabulated Electrochemical Data for Various DNA Origami Tile Designs^a^

	no tile	+ tile A	+ tile B	+ tile C
DPV - δ% peak current (μA)	–8.80 ± 0.06	–0.69 ± 3.45 ns	–28.61 ± 1.61	–13.09 ± 0.04
			*p* ≤ 0.0001	*p* ≤ 0.0001
EIS - δ% *R*_CT_ (Ω)	14.52 ± 0.09	1.83 ± 4.05 ns	82.54 ± 9.61	20.70 ± 0.08
			*p* ≤ 0.0001	*p* = 0.0002

a Mean % change is provided for FE following incubation with a complementary target : tile complex of matched concentrations.

### Electrochemical Performance of a DNA Origami Tile-Enhanced Biosensor

**Figure 4:**
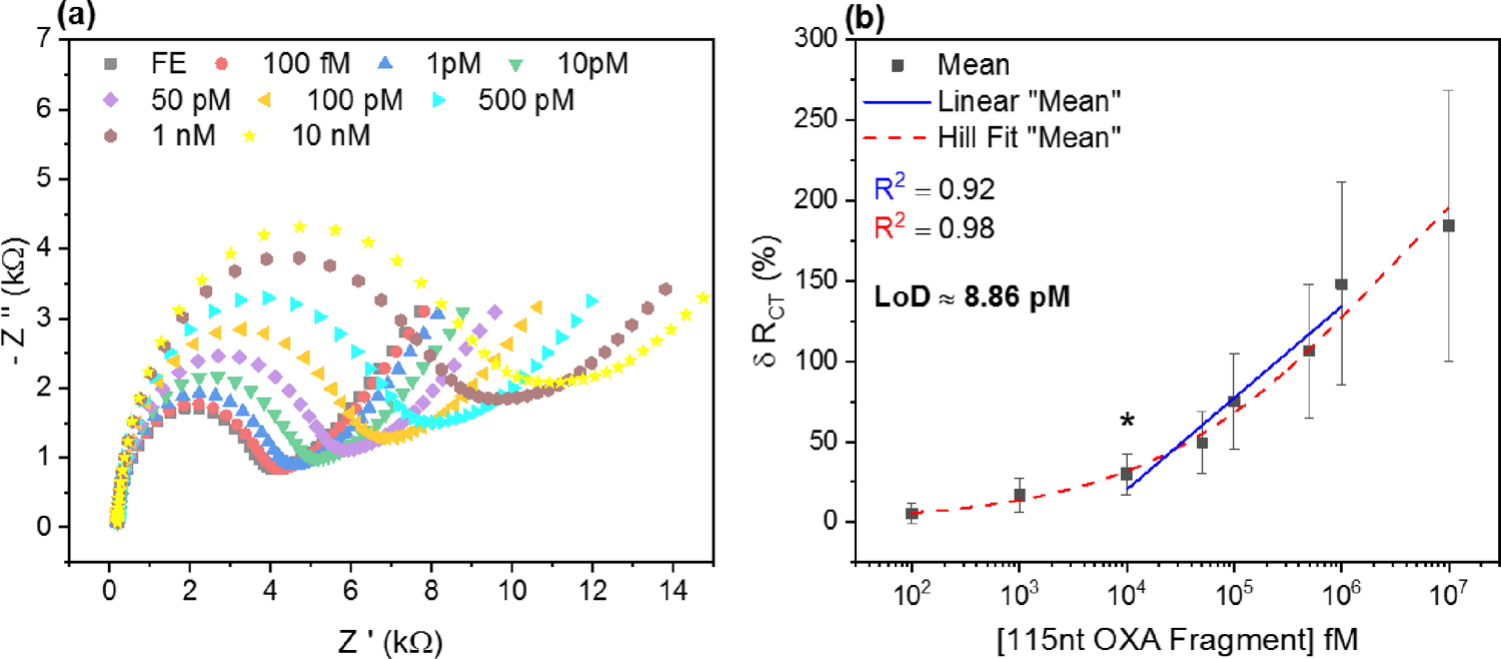
Electrochemical sensor performance with DNA origami tile amplification. (a) Nyquist plot of averaged EIS measurements in response to varying concentrations of the complementary target (115 nt OXA fragment) and tile B at a fixed concentration of 50 pM. (b) Mean *R*_CT_ plotted against a varying concentration of the complementary target (*n* = 4 PGE). Error bars = SD.

With an appropriate design confirmed, it was next necessary to assess the performance of our approach by investigating its response to complementary targets. The decline in redox events in the cell can be associated with the accumulation of local negative charge densities forming through the successful tethering of the large origami tile to the immobilized probe, by the connecting complementary 115 nt OXA fragment. The net effect is the electrostatic repulsion of the redox couple from the functionalized electrode, inhibition of redox mediation, and subsequent growth in *R*_CT_ (Figure 4a).

In this experiment, much of the data fall in a sigmoidal curve, which can again be well-fitted by the Hill equation (eq 1). This is evidenced in Figure 4b with a strong correlation coefficient of 0.99. While there are many descriptions of how the LoD is determined and quantified in the literature, a general description would be the minimum concentration of the analyte that will induce an instrumental signal change (in this case *R*_CT_) that is significant against the pretarget or blank condition. By using eq 2, we can derive an estimated LoD at 8.86 pM. This is supported by *t*-test analysis of experimental data, with significance in the mean signal change first noted following incubation with 10 pM of a target. This provides strong evidence to substantiate the theory of a DNA origami tile serving as an electrochemical signal amplifier. This apparatus has a linear working range between 10 pM and 1 nM, spanning two orders of magnitude. While the lower limit of the working range is three orders of magnitude lower than a conventional pDNA biosensor design, the working range is tighter. We theorize this to be a function of the size of the tile. As such, the electrode surface is quickly saturated, as low target concentrations are sufficient to effectively cross-link these structures to the immobilized probe and induce dramatic interfacial properties.

Interdevice variability in the FE condition is high, though this is a common observation in SAM formation.^12,56^ The notion of low probe DNA coverage contributing to uniform monolayers is perhaps an oversimplification. This is increasingly apparent with reporting of heterogeneous SAM formation^9,57^ and clustering of tightly spaced probes at <10 nm in distance.^58^ Despite this, target hybridization efficiencies are still high and even suggested unexpectedly to be supported by regions of dense probe clustering.^58^ Emerging methods for controlling the distance of neighboring immobilized probes and the incidence of clustering on gold electrodes through electrodeposition are prevalent in the literature.^59−62^ However, as no method of controlling the specific confirmation of the probe spacing has been employed here, the particular degree of uniformity in probe spacing cannot be confirmed. This would be expected to produce a degree of variability in the electrochemical characterization of the functionalized electrode condition. Equally, a contribution to the mean peak current/*R*_CT_ variation in the FE condition may occur from other electrochemical parameters, including real working electrode areas, and cell positioning. This was previously highlighted in Figure 2a, where the box plot reported a large variation in peak current following functionalization across a large sample size (*n* = 42). However, collating the mean percentage change in *R*_CT_ produces consistency in the trend for each electrode and allows for quantitative estimations of the sensor LoD and working range. Therefore, we can conclude a sensitivity enhancement of complementary target detection, supported by our DNA origami tile amplification method (Table 2).

**Table 2:** Tabulated Electrochemical Performance Metrics for a Conventional pDNA Biosensor Design and Our DNA Origami Tile Amplification Strategy

conventional pDNA design	DNA origami amplification
working range: 10 nM–1 μM+	working range: 10 pM–1 nM
LoD: 3.22 nM	LoD: 8.86 pM

### Confirming the Mechanism of Electrochemical Response with a Noncomplementary Target

**Figure 5:**
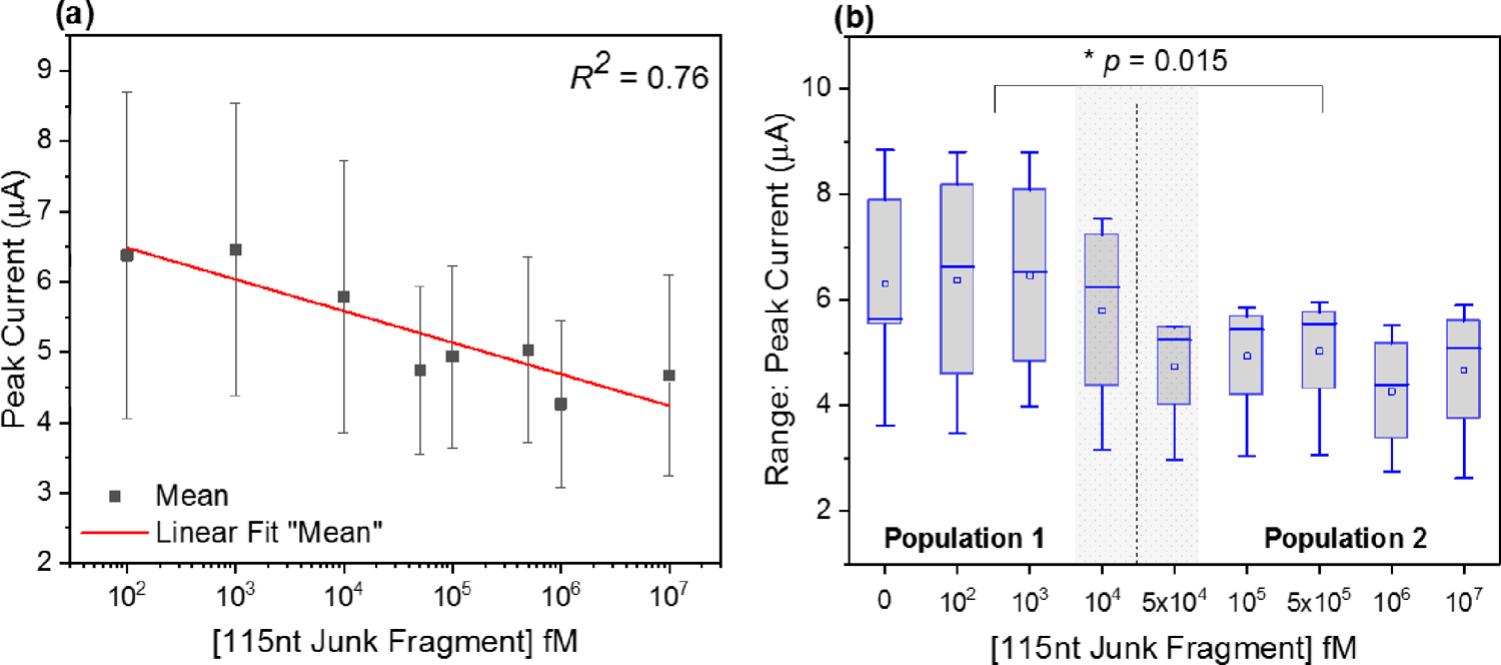
Electrochemical response to a noncomplementary target. (a) Mean peak current response to varying concentrations of a noncomplementary target (115 nt junk fragment) and tile B at a fixed concentration of 50 pM. (b) Box plot of the DPV mean peak current plotted against a varying concentration of a noncomplementary target. The dashed line denotes the division of the data set into two distinct populations. The gray shaded region corresponds to an estimated threshold of nonspecific interactions contributing to an electrochemical signal change. *n* = 4 PGE. Error bars = SD.

With known issues of sensor drift, directly associated with time-dependent alkanethiol SAM reorganization, or degradation of the bioelectric interface through electrochemical interrogation,^10,63−65^ it was necessary to consider if such factors may be influencing the electrochemical responses observed in Figure 4. To do so, an experiment was carried out, incorporating a noncomplementary target of 115 nt length (115 nt junk fragment, see Supporting Information Table S1), designed to have neither a recognition site for the immobilized probe nor the solution-based tile. In Figure 5a,b, the mean peak current is reported in response to incubation with increasing concentrations of the noncomplementary target and a fixed concentration of tile B.

The scatter plot in Figure 5a allows for the fitting of the experimental data to assess whether a linear region is present that could be attributed to concentration-dependent nonspecific DNA interactions or the reorganization effects reported by Piper *et al*.^65^ Fitting of the data is poor with a coefficient correlation of 0.78 across the experimental range and indicative of no sporadic layer organization that contributes solely to a significant decline in peak current. This is better reflected in Figure 5b, where peak current data for each condition are provided as a box plot. This allows for a determination of two significantly distinct populations within the data, denoted 1 and 2 in Figure 5b and separated by the gray dashed line. We suggest two possible phenomena responsible for this deviation in mean peak current. First, the noncomplementary target has reached a concentration whereby nonspecific interactions with the underlying SAM are sufficient to induce a significant step change in mean peak current. Second, tile B has a weak affinity for the monolayer. Successive incubations of the functionalized transducer in 50 pM of tile B result in changing interfacial properties of the bioelectric surface, with an inappropriate immobilization of the tile. However, the magnitude of the signal change attributable to nonspecific interactions is markedly less than that associated with DNA origami tile amplification reported in Figure 4b. To further confirm the benefit of this method of signal amplification by origami nanostructures, the system was interrogated in a complex media containing a high DNA load.

### Specificity of the DNA Biosensor Design

**Figure 6:**
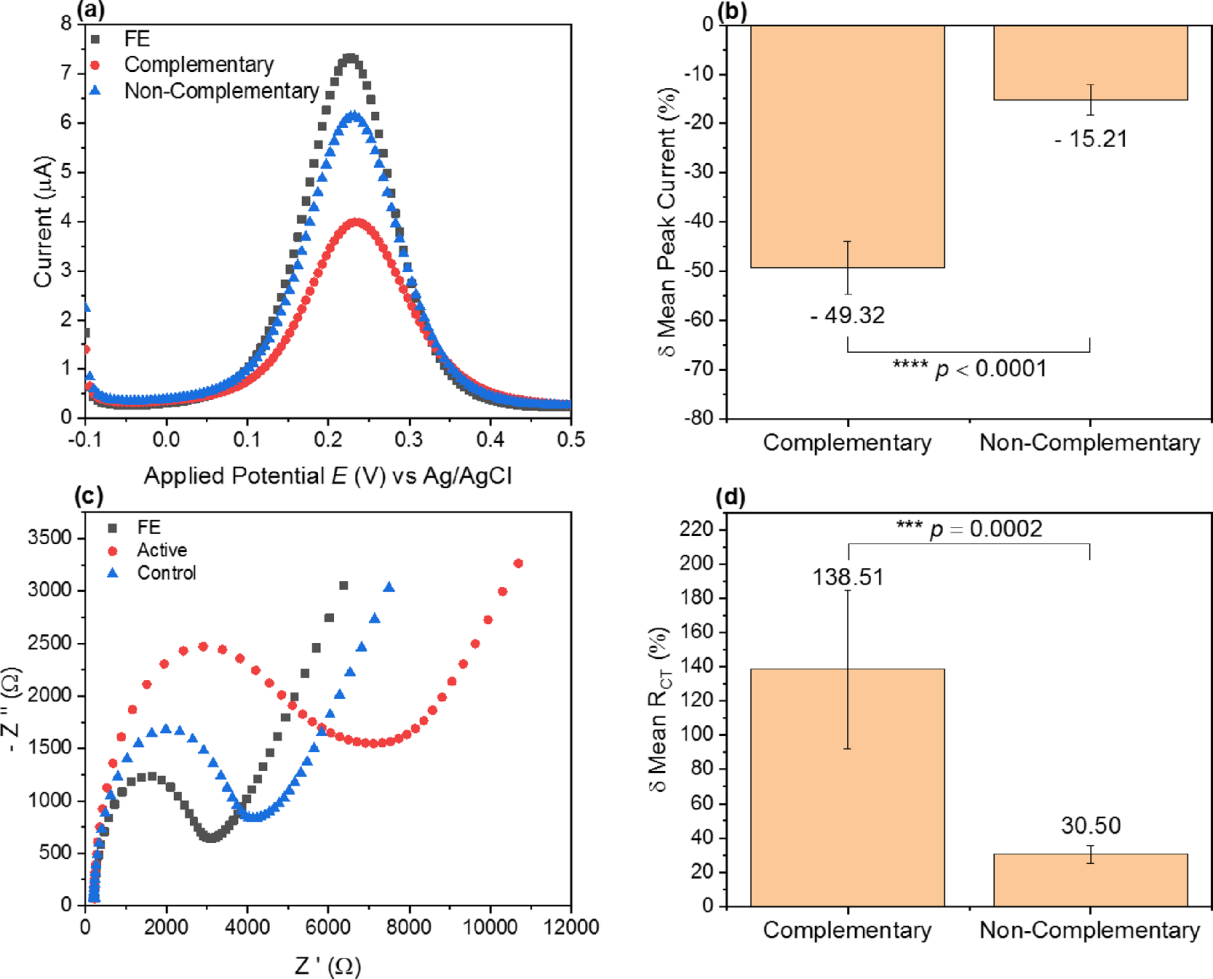
Electrochemical response of sensor design to complementary and noncomplementary targets in a complex media. (a) Mean DPV signal response to 100 pM of a complementary target (115 nt OXA fragment) and 100 pM of a noncomplementary target (115 nt junk fragment) against tile B at a fixed concentration of 50 pM. (b) Mean percentage change for peak current following complementary and noncomplementary target incubation. (c) Mean Nyquist plot response to 100 pM of a complementary target and 100 pM of a noncomplementary target against tile B at a fixed concentration of 50 pM. (d) Mean percentage change for *R*_CT_ following complementary and noncomplementary target incubation. *n* = 3 PGE with duplicate measurement. Error bars = SD.

To validate the hypothesized sensor mechanism of action, an experiment was undertaken subjecting functionalized electrodes to tile B at a concentration of 50 pM and either of the two target sequences used previously in this study: the complementary sequence, the 115 nt OXA fragment, and a randomly generated noncomplementary sequence of 115 nt length, the 115 nt junk fragment. Additionally, the sensing apparatus is challenged by undertaking the assay in a complex media. Here, a complex media is established by spiking components of a commercially available DNA origami folding kit (Tilibit Nanosystems) to each sample, thus producing a high background nonspecific DNA load on the sensor. Confirmation of the sensor mechanism is provided in Figure 6.

Figure 6a displays peak amplitude depression for both the complementary and noncomplementary target incubations. However, the magnitude of peak reduction is significantly larger for the complementary target. This is documented in Figure 6b with the respective percentage change of mean peak currents contrasted between both targets, with a high degree of significant difference noted (****p < 0.0001). This is furthered by the data of impedimetric measurements presented in the bottom panel of Figure 6. The Nyquist plot of Figure 6c shows the characteristic growth of the semicircle region associated with increasing impedance. Again, this is common to both complementary and noncomplementary targets; however, the magnitude of the signal change is significantly greater for the complementary target. The specific mean percentage change of *R*_CT_ is given in Figure 6d with the increase in charge transfer resistance significantly enhanced with a complementary target (***p = 0.0002).

This is a particularly noteworthy result, given the large background content of DNA in solution, spiked to further challenge the sensor design. Constituent components of the sample solution include either the complementary or noncomplementary targets at 100 pM, tile B at 50 pM, and the necessary concentrations of all reagents required for the assembly of a commercially available DNA origami nanostructure provided by Tilibit Nanosystems. The details of the reaction mix from Tilibit Nanosystems are provided in Supporting Information Table S4. The sensor is therefore interrogated with a working concentration of the p7249 scaffold at a concentration of 1.5 nM (15× greater than the target concentration) and the staple strands at a concentration of 76 nM (760× greater than the target concentration).

With a high background DNA concentration, the impact of nonspecifics was theorized to be significant. All incubation steps are undertaken at a temperature of 37 °C, and hybridization of regions of the scaffold and staples was expected to produce incomplete secondary structures. Consequently, the incidence of nonspecific interactions between any such structure or inherent component, with any of the immobilized probe, target, or tile, may contribute to the magnitude of the signal change.

The electrochemical data given in Figure 6 would support this theory, with a meaningful signal change associated with the noncomplementary target experiment. However, the ability to discriminate with a high power of significance between complementary and noncomplementary target experimentation corroborates previous data supporting signal amplification by a DNA origami tile. The contribution of nonspecific DNA interactions is commonly observed in biosensor literature, and there are multiple avenues to explore in minimizing their input enhancing the stringency of washing stages to strip nucleotides adsorbed on exposed gold by ion-induced dipole binding^66^ or through hybridization with partial sequence complementarity. Introduction of microfluidic control may further the consistency of such washing stages and provide a reduction in the manual processing step count, better tailoring the system to a PoC setting. Moreover, the underlying SAM formed on the transducer surface can be reinvestigated with considerations raised by Shaver *et al.*, with modifications to the hydrophobicity of the constituent alkanethiols contributing to enhanced SAM stability,^10^ or the charge characteristics of certain end group moieties.^67^ Improvements in the underlying bioelectric properties may provide a greater magnitude in signal amplification possible through our approach and advance the applicability of a SAM-based biosensor for a PoC device. Finally, a crucial factor to be aware of when interpreting the data presented in this study is error and more specifically electrochemical error, which arises from a number of places, including the fact that each electrode must be cleaned and polished prior to reuse, the irreproducible nature of SAM formation, and the high sensitivity of the impedance method to surface conditions. Together, these factors compound to produce data with significant error bars, especially when compared to other analytical techniques. These errors are present throughout and represent measured electrode variation. However, the fitting techniques used and the control experiments employed mean that the conclusions drawn from the study are valid.

## Conclusions

This study successfully introduces a DNA origami nanostructure to aid in boosting the electrochemical signal gain associated with target hybridization. In harnessing the high programmability of the origami method, it has been possible to create a sandwich assay, where a target oligonucleotide hosting the *bla*OXA-1 β-lactamase gene serves to effectively cross-link the nanostructure to a functionalized electrode, and significantly modify surface interfacial properties. As such, simple label-free electrochemical methods allow for enhanced detection limits of two orders of magnitude for DNA sequences encoding antimicrobial resistance, without the requirement for complex surface modifications or enzymatic support. In addition, this sensor design proves effective in discriminating between complementary and noncomplementary targets in a complex media, rich in nucleic acids confirming the power of its specificity. With the ever-declining cost of oligonucleotide synthesis, simplicity, and elegance of origami design, we report these findings as a promising platform for signal amplification with a host of nucleic acid targets and of direct relevance to tackling strict sensitivity requirements in PoC devices.

## Supporting Information Available

DNA origami-related materials, caDNAno design, list of replaced staples, DNA origami assembly and purification protocols, AGE and TEM protocols, additional TEM images, DNA sequences for electrochemical assays, materials for electrochemical measurements, measurement scripts, and equivalent circuit for data fitting (PDF)

## Notes

A patent entitled “Electrochemical Biosensor” (PE962296GB) has been filed based on this work.
