# Supplementary material for: Signal Amplification in Electrochemical DNA Biosensors Using Target-Capturing DNA Origami Tiles
Source: ACS Sens. 2023 Mar 13;8(4):1471–80. doi: 10.1021/acssensors.2c02469 (PMC10152479; doi:10.1021/acssensors.2c02469)
Supplement: Supplementary file 1 — se2c02469_si_001.pdf [file se2c02469_si_001.pdf]

Supporting Information

Signal Amplification in Electrochemical DNA Biosensors Using Target-Capturing DNA Origami Tiles

Paul Williamson,[†] *Petteri Piskunen,*[‡] *Heini Ijäs,*[‡,§] *Adrian Butterworth,*[†] *Veikko Linko,**[‡,//,¥] *and Damion K. Corrigan**[†,±]

[†] Department of Biomedical Engineering, University of Strathclyde, Glasgow G1 1QE, United Kingdom

[‡] Biohybrid Materials, Department of Bioproducts and Biosystems, Aalto University, 00076 Aalto, Finland

[§] Ludwig-Maximilians-University, Geschwister-Scholl-Platz 1, 80539 Munich, Germany

[//] LIBER Center of Excellence, Aalto University, 00076 Aalto, Finland

[¥] Institute of Technology, University of Tartu, Nooruse 1, 50411 Tartu, Estonia

[±] Department of Pure & Applied Chemistry, Thomas Graham Building, 295 Cathedral Street, University of Strathclyde, Glasgow G1 1XL, United Kingdom

Corresponding Authors:
*Email: veikko.pentti.linko@ut.ee
*Email: damion.corrigan@strath.ac.uk

Table of Contents

1. DNA Origami Tile Materials 2

2. Design and Capture Strands of the DNA Origami Tile 3
Capture Strands of Tile B 4
Capture Strands of Tile C 4

3. DNA Origami Assembly 5

4. DNA Origami Purification 5

5. Agarose Gel Electrophoresis (AGE) 5

6. Transmission Electron Microscopy (TEM) 6

7. Materials for Electrochemical Measurements 8

8. Electrode Preparation, Electrochemical Measurement, Functionalization, and Target Detection 9

9. Supporting Information References 13

1. DNA Origami Tile Materials

All staple strands constituting the used DNA origami tiles were purchased from Integrated DNA Technologies and the employed M13mp18 scaffold strand was obtained from Tilibit Nanosystems. 50× stock TAE (Tris/acetic acid/ethylenediaminetetraacetic acid (EDTA)) buffer was purchased from Thermo Fisher Scientific (Finland) and molecular grade agarose from Meridian Bioscience (Ohio, US). All other chemicals required in the DNA origami assembly, purification and characterization were sourced from Merck/Sigma-Aldrich (Finland). Milli-Q deionized water was used in all procedures. DNA origami annealing was carried out in a Biometra T-Gradient thermocycler. Agarose gel electrophoresis was performed using a BioRad Mini-Sub Cell GT System with a BioRad PowerPac Basic power supply and imaged with a Bio-Rad ChemiDoc MP Imaging System. Concentrations were measured with a BioTek Eon Microplate UV/Vis spectrophotometer and a Take3 micro-volume plate. Transmission electron microscopy (TEM) sample grids (FCF400-CU) were sourced from Electron Microscopy Sciences, treated with a NanoClean 1070, Fischione Instruments plasma cleaner and imaged using a FEI Tecnai 12 TEM.

2. Design and Capture Strands of the DNA Origami Tile

The full caDNAno[S1] design of the DNA origami tile[S2] is shown in **Figure S1**. The modification strands are highlighted in **turquoise** and **pink**, for the front and back sides of the tiles respectively.

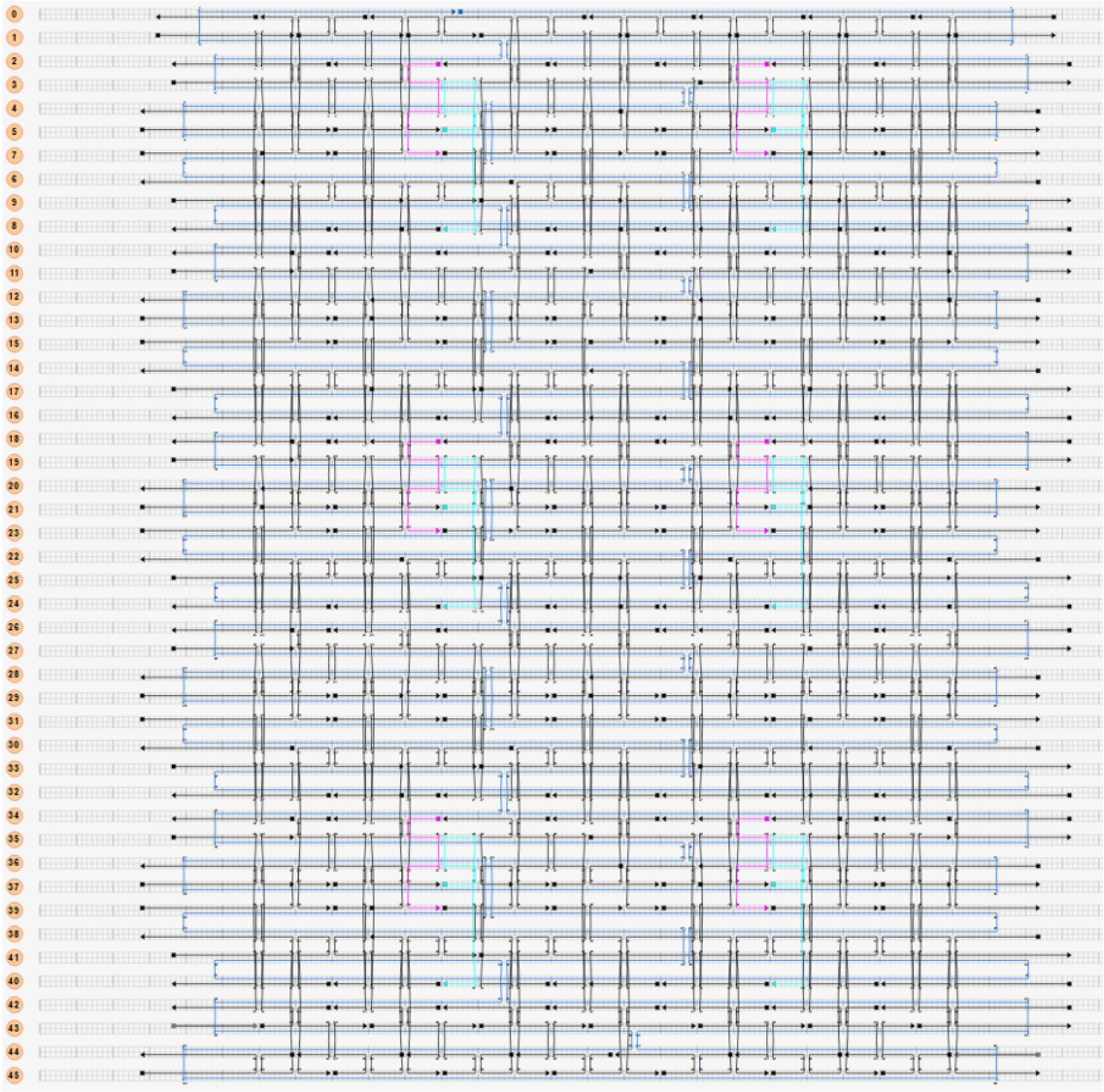

Figure S1. caDNAno design of the DNA origami tile.

The following extended strands are used to replace corresponding core staples in the DNA origami tile to create Tiles B and C. The core staple segment of the replacement strands are highlighted in **turquoise** and **pink** (See **Figure S1**), while blue denotes the used capture sequence. "tttttt" is a poly-T_6 spacer. Sequences are given from 5' to 3' (the first column indicates the start and end positions of the strand in the caDNAno design).

Capture Strands of Tile B

position	sequence 5' – 3'
37[77]<br>-40[77]	AAAAGGCATGGGATTGAGTTAAGCCAGCttttttTTGTCTTCGTACCGAGCTTTCATCGAATTTTTA
21[140]<br>-24[140]	TTAATTGTAACAGTTTTGACCATTTAATttttttTTGTCTTCGTACCGAGCTTTCATCGAATTTTTA
5[77]<br>-8[77]	TGCAGGTCCAGCTGTACAGGGGGCCAACttttttTTGTCTTCGTACCGAGCTTTCATCGAATTTTTA
5[140]<br>-8[140]	TTTCCTGTAATGAGCTGCCCGGAAATGGttttttTTGTCTTCGTACCGAGCTTTCATCGAATTTTTA
37[140]<br>-40[140]	TTAAACAGCTTGCACCCTCAGGACGGAAttttttTTGTCTTCGTACCGAGCTTTCATCGAATTTTTA
21[77]<br>-24[77]	GATTAGAGCGGATTCAAGAAAATCATAAttttttTTGTCTTCGTACCGAGCTTTCATCGAATTTTTA

Capture Strands of Tile C

37[77]<br>-40[77]	AAAAGGCATGGGATTGAGTTAAGCCAGCttttttTTGTCTTCGTACCGAGCTTTCATCGAATTTTTA
21[140]<br>-24[140]	TTAATTGTAACAGTTTTGACCATTTAATttttttTTGTCTTCGTACCGAGCTTTCATCGAATTTTTA
5[77]<br>-8[77]	TGCAGGTCCAGCTGTACAGGGGGCCAACttttttTTGTCTTCGTACCGAGCTTTCATCGAATTTTTA
5[140]<br>-8[140]	TTTCCTGTAATGAGCTGCCCGGAAATGGttttttTTGTCTTCGTACCGAGCTTTCATCGAATTTTTA
37[140]<br>-40[140]	TTAAACAGCTTGCACCCTCAGGACGGAAttttttTTGTCTTCGTACCGAGCTTTCATCGAATTTTTA
21[77]<br>-24[77]	GATTAGAGCGGATTCAAGAAAATCATAAttttttTTGTCTTCGTACCGAGCTTTCATCGAATTTTTA

18[139]<br>-23[139]	AGTAGCAGATTTAGTGATTCCTAGAATCttttttTTGTCTTCGTACCGAGCTTTCATCGAATTTTTA
2[76]<br>-7[76]	AAAGCGAGCGCCGCGCGAAAGGGCCTCTttttttTTGTCTTCGTACCGAGCTTTCATCGAATTTTTA
34[139]<br>-39[139]	AAAATACATCGTCAGGGAGTTCGGAATAttttttTTGTCTTCGTACCGAGCTTTCATCGAATTTTTA
2[139]<br>-7[139]	TTTCTTTGCGCTCATGAGCTAGTGTTTTttttttTTGTCTTCGTACCGAGCTTTCATCGAATTTTTA
18[76]<br>-23[76]	ATTGCTTCAAACATGCATCAAATTATAGttttttTTGTCTTCGTACCGAGCTTTCATCGAATTTTTA
34[76]<br>-39[76]	GCGCATTCAAGAATTTTGCTAAGTAAATttttttTTGTCTTCGTACCGAGCTTTCATCGAATTTTTA

3. DNA Origami Assembly

The DNA origami tiles were assembled by first mixing a ~10× molar excess of synthetic staple strands with a circular 7,249-nucleotide (nt) long M13mp18 scaffold strand (p7249) in 2.5× folding buffer (FOB: TAE buffer supplemented with $MgCl_2$ and NaCl). The resulting solution contained 20 nM of scaffold and ~200 nM of each staple strand in 1× FOB (1× TAE (40 mM Tris, 19 mM acetic acid, 1 mM ethylenediaminetetraacetic acid (EDTA)) with 20 nM $MgCl_2$ and 5 nM NaCl, pH ~8.5). A list of all replaced staples is given above. The mixtures were heated to 90 ℃ and slowly annealed according to the following thermal ramp: Cooling from 90 ℃ to 70 ℃ at –1.5 ℃/min, from 70 ℃ to 60 ℃ at –0.75 ℃/min, and from 60 ℃ to 27 ℃ at –0.05 ℃/min.

4. DNA Origami Purification

After annealing, the now folded DNA origami were purified using polyethylene glycol (PEG) precipitation.[S3] Here, the unpurified origami were diluted to ~5 nM concentration with 1× FOB and mixed 1:1 (v/v) with the PEG precipitation buffer (1× TAE, 15% (w/v) PEG 8000, 505 mM NaCl). The mixture was then centrifuged at 14,000× *g* for 30 min at room temperature. After centrifugation, the supernatant was removed by pipetting and the remaining DNA origami pellet was dissolved in its original volume of 1× FOB. The solution was then incubated overnight at room temperature to resuspend the DNA origami tiles. Finally, the concentrations of the purified DNA origami solutions were determined with an UV/Vis spectrophotometer.

5. Agarose Gel Electrophoresis (AGE)

AGE was used to verify the integrity of the DNA origami tiles (main manuscript **Figure 1b**). A 2% (w/v) agarose gel was prepared in 1× TAE with a 11 mM $MgCl_2$ concentration and a 0.46 µg/mL ethidium bromide staining. 10 µL aliquots were prepared from each of the investigated DNA origami samples by diluting them to a uniform 15 nM concentration with 1× FOB. Then, 2 µL of 6× gel loading solution was added to each aliquot and the samples were loaded into the gel. Similarly prepared 15 nM M13mp18 scaffold was used as the reference band. The gel was run for 45 min at 90 V in an ice bath with 1× TAE containing 11 mM of $MgCl_2$ as the running buffer. A Bio-Rad ChemiDoc MP Imaging System was used to image the gel under ultraviolet light.

6. Transmission Electron Microscopy (TEM)

The fabricated DNA origami tiles were also imaged with TEM (main manuscript **Figure 1b**), based on a sample preparation protocol by Castro *et al.*[S4] A 3 µL droplet of ~20 nM origami solution was deposited on an O_2 plasma cleaned (20 s flash) formvar carbon-coated copper TEM grid and incubated for 1 min. After incubation, the droplet was drained with a piece of filter paper and sequentially negatively stained with 2% (w/v) uranyl formate that contained 25 mM of NaOH. The grid was first immersed in a smaller 5 µL uranyl formate droplet, immediately drained with filter paper and then immersed in a larger 20 µL droplet before incubating for 45 s. After incubation, the grid was once more blotted with filter paper and left to completely dry in ambient conditions for at least 30 min before imaging with TEM. For imaging, a 120 kV acceleration voltage was used. See the **Figures S2-S4** for additional TEM images of all used DNA origami tile variants.

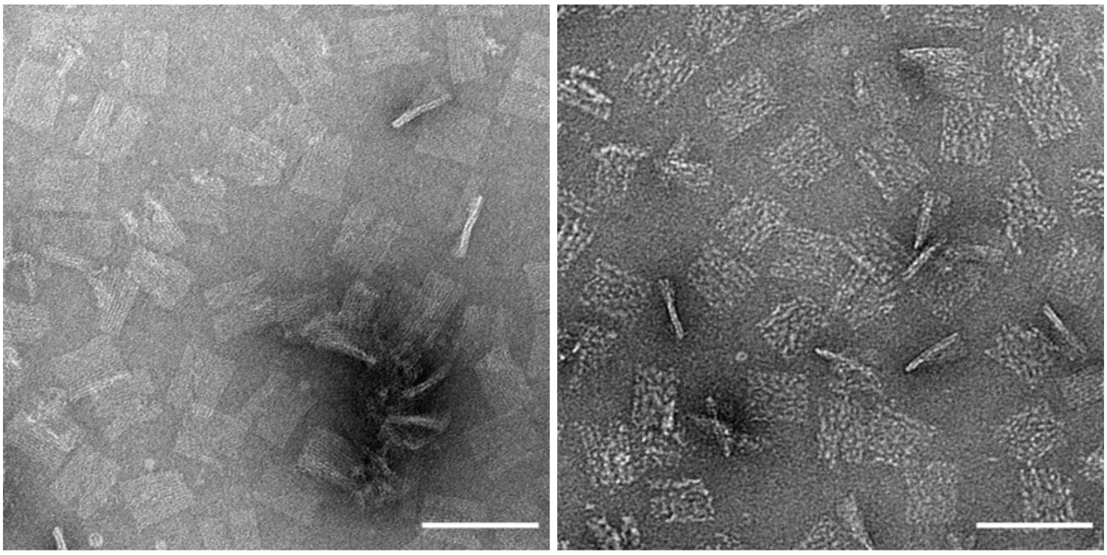

Figure S2. Tile A, no capture strands. Scale bars are 100 nm.

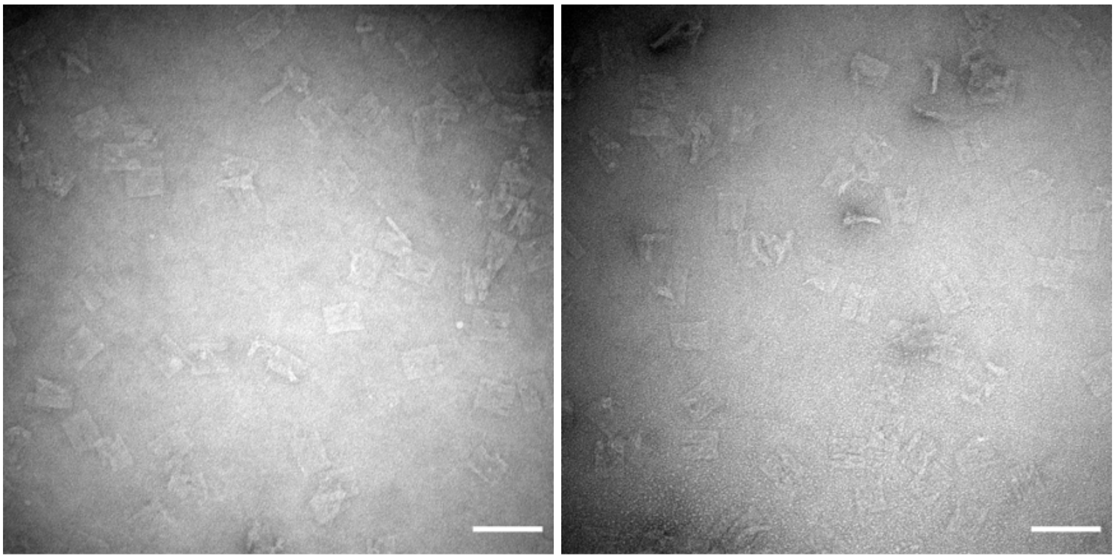

Figure S3. Tile B, capture strands on one side. Scale bars are 100 nm.

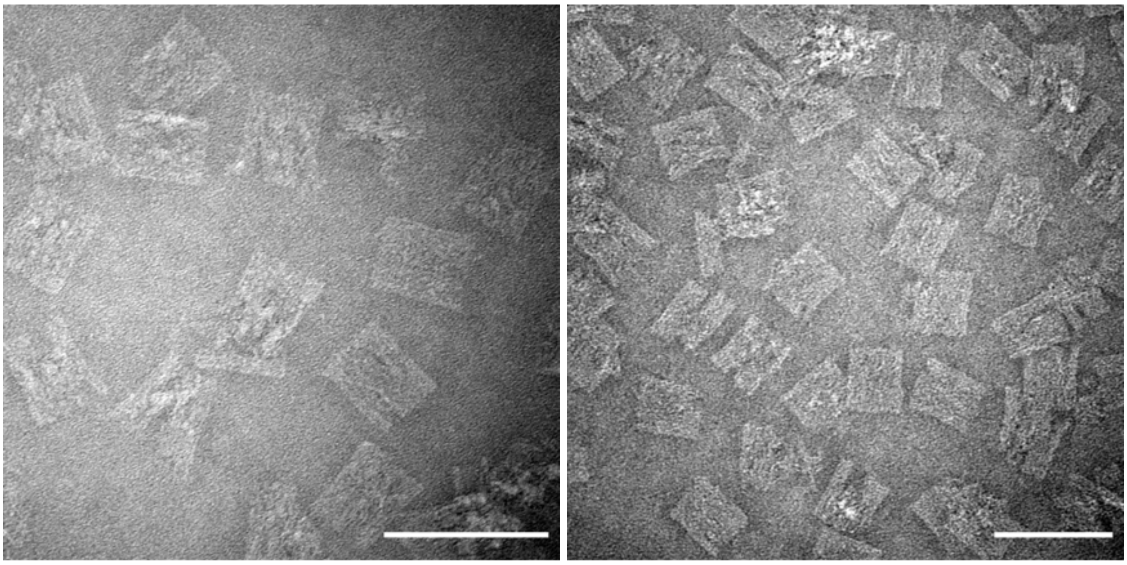

Figure S4. Tile C, capture strands on both sides. Scale bars are 100 nm.

7. Materials for Electrochemical Measurements

Table S1. Oligonucleotides for sensor construction and assay development (the complementary sequences are underlined).

	**sequence 5’ – 3’**
**OXA probe**	**GGTGTTTTCTATGGCTGAGTTTTTAACTGGGAG**
**115-nt OXA Fragment**	**AACAGAAGCATGGCTCGAAAGTAGCTTAAAAATTTCACCAGAAGAACAAATTCAATTCCTGCGTA AAATTATTAATCACAATCTCCCAGTTAAAAACTCAGCCATAGAAAACACC**
**115-nt Junk Fragment**	**TATTACTTTTGCCTCAACGGCTCCTGCTTTCGCTGAAACCCAAGACAGGCAACAGTAACCGCCTT TTGAAGGCGAGTCCTTCGTCTGTGACTAACTGTGCCAAATCGTCTTCCAA**

Table S2. Buffers used in this study.

**Buffer**	**Composition**
Piranha Solution	18 M H_2SO_4 + 30 % H_2O_2 at a 3:1 (v/v) ratio
TM Buffer (pH 8)	Tris-HCl (10 mM) + 50 mM $MgCl_2 \cdot 6H_2O$
TM Buffer (pH 8) + TCEP	50 µM TCEP in Tris-HCl (10 mM) + 50 mM $MgCl_2 \cdot 6H_2O$
Redox Buffer	2 mM Potassium Ferricyanide / Potassium Ferrocyanide in 1x PBS
Rinse Buffer	1× PBS

8. Electrode Preparation, Electrochemical Measurement, Functionalization, and Target Detection

Electrode Preparation and Electrochemical Measurement. Appropriate cleaning is required to achieve conformity in polycrystalline gold electrode (PGE) surfaces, and the removal of immobilized organics and contaminants. Mechanical polishing was first undertaken to produce a near mirror finish via a series of decreasing alumina slurry diameters from 1 μm to 0.03 μm, on microcloths of varying roughness, with sonication in isopropanol (IPA) for 2 min between each polishing step. Polishing occurred in a figure of eight motion for a duration of two min per electrode. Stripping of organics was attained by immersion of the gold surfaces in hot piranha (H_2SO_4 and H_2O_2 3:1 (v/v)) for 15 min. Finally, electrochemical cleaning was undertaken by repeated cyclic voltammetry (**Table S3**) in 0.1 M H_2SO_4 at 0.1 V/s, with a potential window of –0.1 to 1.6 V until a stable reduction peak was observed in the voltammogram (10–15 scans). It was necessary to confirm the effectiveness of the cleaning protocol with subsequent electrochemical interrogation of each PGE by Faradaic methods.

Measurement Script 1 was used to determine if key analytical tools of Peak Current (A) from differential pulse voltammetry (DPV) and R_{CT} (Ω) from electrochemical impedance spectroscopy (EIS) lie within a consistent range for PGE immersed in the redox buffer. Details of the measurement script are provided in **Table S3.**

Table S3. Measurement Scripts for electrochemical interrogation.

**Cleaning CV 1**	**Measurement Script 1**	**Measurement Script 2**
For the stripping of contaminants from PGE	*Performance characterisation*	*Determining surface coverage*
**Cyclic Voltammetry**	**DPV**	**Chrono Colometry** (Δt > 1 ms)
**Potential Window:**	**Potential Window:**	**Apply Potential:**
-0.1 V to 1.6 V	-0.1 V to 0.5 V	0 V

**Scan Rate:**	**Step:**	***Record Signal:***
0.1 V/s	0.005 V	**Duration:**
		1 s
**Number of scans:**		**Interval Time:**
10	**SWV**	0.0025 s

	**Potential Window:**	**Apply Potential:**
	-0.1 V to 0.5 V	0.15 V
	**Step:**	---------
	0.005 V	***Record Signal:***
	**Frequency (Hz):**	**Duration:**
	25	1 s
		**Interval Time:**
		0.0025 s
	**OCP Determination**	---------
		Apply Potential:
	**Duration:**	*-0.35 V*
	20 seconds	---------
		Record Signal:
	**EIS**	**Duration:**
		2 s
	**Applied Potential:**	**Interval Time:**
	0 V vs OCP	0.0025 s
	**Frequency Range (Hz):**	
	10k – 0.1	
	**N^{O} of Frequencies / decade:**	
	10	

PGE that report mean signals for any one of the above that exist out with 1.5 interquartile range (IQR), were discounted and not carried forward for further experimental work. Electrochemical circuit fitting of Nyquist Data from EIS measurements is required to extract analytical parameters of solution resistance (R_s), charge transfer resistance (R_{CT}), and capacitance (C). The simplified Randles circuit was chosen for circuit fitting of electrochemical data (provided in **Figure S5**). Square Wave Voltammetry (SWV) interrogation of the sensor design was also explored, given its potential for enhancing signal gain reported in literature. However, SWV performance is a direct function of measurement frequency, and pulse amplitude. Both of which require close refinement for specific

probe architectures, monolayer packing densities, and the electron transfer rates of the redox reporter.[S5] To date, we have yet to undertake such a study to explore the electrochemical parameters required to facilitate a sensing enhancement by SWV, and current recorded data shows no meaningful improvement against DPV interrogation. For simplicity, SWV analysis has not been reported here.

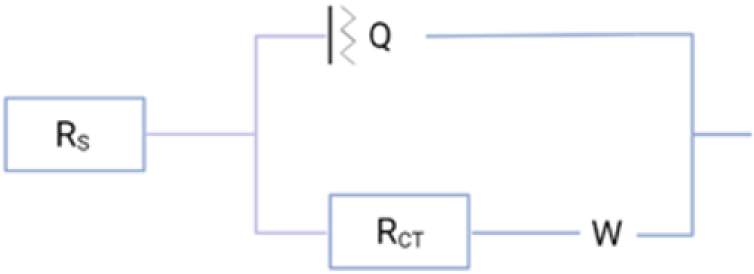

Figure S5. An analogous electrical circuit for EIS data fitting. Solution Resistance (R_s), Charge Transfer Resistance (R_{CT}), Constant Phase Element (Q) and Warburg Element (W).

Electrode Functionalization. After cleaning, electrodes were immersed in ethanol for 3 min, rinsed in Di-H_2O, and then dried under a steady Argon stream. A mixed self-assembled monolayer (SAM) of pDNA and MCP was formed by overnight incubation (18 h) at 37 °C, with electrodes immersed in a solution of 1 µM probe : 10 µM MCP, in excess 50 µM TCEP (Tris (2-carboxyethyl)phosphine hydrochloride). The primary solvent throughout was TM buffer at pH 8 (10 mM Tris-HCl + 50 mM $MgCl_2{\cdot}6H_2O$). Following this step, electrodes are named as functionalized electrodes (FE). With the completion of the functionalization protocol, all electrodes were rinsed in a gentle flow of DI water for 10 s to remove non-specifically adsorbed oligonucleotides from the sensor surface. FE were then dried under a steady stream of argon.

It was necessary to assess the performance of the functionalization protocols. FE were subject to electrochemical interrogation in the redox buffer, following Measurement Script 1. Again, any data point existing out with 1.5 IQR was noted as evidence of abnormal functionalization and this electrode was discounted from further study.

Target Detection. The detection method of this approach is centered around the capture of a DNA origami tile/target complex from solution by an immobilized probe on the electrode surface. As such, it was first necessary to incubate a solution of both Tile A, B, or C and target to allow this complex to form. In this study, the tile was held at a fixed concentration (dependent upon particular experimental aim) against a varying target concentration. This complex was allowed to form by a 30 min incubation

at 37 °C. After which, FE were incubated directly in this solution for a further 30 min at 37 °C. Following all Target incubations, electrodes were rinsed in 1× PBS (phosphate-buffered saline) for 10 s, and gently dried under a steady stream of argon gas. They were then immersed in a redox buffer for the electrochemical characterization of sensing performance using Measurement Script 1 (**Table S3**). To confirm the applicability of this sensor construction in detecting key DNA targets of clinical interest, bacterial DNA sequences central to antimicrobial resistance are employed in assay development. Here, immobilized probes, and capture strands are primer sequences for the amplification of a region of an artificial plasmid attributing to the *bla*OXA-1 β-lactamase gene; encoding extended-spectrum β-lactamases (ESBLs) and resistance to Oxacillin, across a host of gram-negative species. This *bla*OXA-1 β-lactamase gene sequence (OXA Fragment) serves as the complementary target sequence in this study.

Commercially available reaction mixtures were purchased from Tilibit Nanosystems in order to challenge the sensing apparatus: a DNA origami scaffold strand:
https://www.tilibit.com/products/folding-kit-basic-type-p7249-m13mp18,
and staple strands for a cuboid with large aperture:
https://www.tilibit.com/collections/prefabricated-structures/products/cuboid-with-large-aperture.
This mixture was not heated, to allow for minimal secondary structure formation. The below mixture (**Table S4**) was spiked during incubation stages, to provide a high concentration of background DNA.

Table S4. Reaction mixture for Tilibit Nanosystems assemblies.

**Component**	**Volume added (µL)**
**10× folding buffer**	10
**100 nM single-stranded scaffold DNA (type p7249)**	6
**200 mM $MgCl_2$**	20
**475 nM staple mixture (Cuboid with large aperture)**	64

9. Supporting Information References

(S1) Douglas, S. M.; Marblestone, A. H.; Teerapittayanon, S.; Vazquez, A.; Church, G. M.; Shih, W. M. Rapid Prototyping of 3D DNA-Origami Shapes with caDNAno. *Nucleic Acids Res*. **2009**, *37* (15), 5001–5006. https://doi.org/10.1093/nar/gkp436.

(S2) Julin, S.; Nonappa; Shen, B.; Linko, V.; Kostiainen, M. A. DNA-Origami-Templated Growth of Multilamellar Lipid Assemblies. *Angew. Chem. Int. Ed*. **2021**, *60* (2), 827–832. https://doi.org/10.1002/anie.202006044.

(S3) Stahl, E.; Martin, T. G.; Praetorius, F.; Dietz, H. Facile and Scalable Preparation of Pure and Dense DNA Origami Solutions. *Angew. Chem., Int. Ed*. **2014**, *53* (47), 12735–12740. https://doi.org/10.1002/anie.201405991.

(S4) Castro, C. E.; Kilchherr, F.; Kim, D.-N.; Shiao, E. L.; Wauer, T.; Wortmann, P.; Bathe, M.; Dietz, H. A Primer to Scaffolded DNA Origami. *Nat. Methods* **2011**, *8*, 221–229. https://doi.org/10.1038/nmeth.1570.

(S5) Dauphin-Ducharme, P.; Plaxco, K.W. Maximizing the Signal Gain of Electrochemical-DNA Sensors. *Anal. Chem*. **2016**, *88* (23), 11654–11662. https://doi.org/10.1021/acs.analchem.6b03227.
